# Extraction, structural characterization, and antioxidant activity of polysaccharides derived from *Arctium lappa* L.

**DOI:** 10.3389/fnut.2023.1149137

**Published:** 2023-03-21

**Authors:** Dandan Gao, Hong Chen, Haixing Li, Xuhua Yang, Xingchen Guo, Yuxuan Zhang, Jinpu Ma, Jutian Yang, Shuwen Ma

**Affiliations:** ^1^China-Malaysia National Joint Laboratory, Biomedical Research Center, Northwest Minzu University, Lanzhou, China; ^2^College of Life Sciences and Engineering, Northwest Minzu University, Lanzhou, China; ^3^Sino-German Joint Research Institute, Nanchang University, Nanchang, China; ^4^Taizishan Ecosystem Observatory of Carbon Neutralization, Northwest Minzu University, Lanzhou, China

**Keywords:** *Arctium lappa* L., polysaccharides, aqueous two-phase system, response surface methodology, antioxidant activity

## Abstract

**Introduction:**

*Arctium lappa* L. root has high nutritional and medicinal values and has been identified as a healthy food raw material by the Ministry of Health of the People's Republic of China.

**Methods:**

In the present study, an aqueous two-phase system (ATPS) of polyethylene glycol (PEG)-(NH_4_)_2_SO_4_ was used to extract *Arctium lappa* L. polysaccharides (ALPs) from the *Arctium lappa* L. roots, the optimal extraction conditions of crude ALPs were optimized by using the single-factor experiment and response surface methodology. The structure and composition of ALPs were determined by fourier transform infrared spectroscopy (FTIR), scanning electron microscopy (SEM), and high-performance liquid chromatography (HPLC). At the same time, the antioxidant activity of ALPs was investigated by *in vitro* antioxidant experiment.

**Results:**

The optimized extraction parameters for extraction ALPs were as follows: the PEG relative molecular weight of 6,000, a quality fraction of PEG 25%, a quality fraction of (NH_4_)_2_SO_4_ 18%, and an extraction temperature of 80°C. Under these conditions, the extraction rate of ALPs could reach 28.83%. FTIR, SEM and HPLC results showed that ALPs were typical acidic heteropolysaccharides and had uneven particle size distribution, an irregular shape, and a rough surface. The ALPs were chiefly composed of glucose, rhamnose, arabinose, and galactose with a molar ratio of 70.19:10.95:11.16:6.90. In addition, the ALPs had intense antioxidant activity *in vitro* with IC_50_ values in the ·OH radical (1.732 mg/ml), DPPH radical (0.29 mg/ml), and superoxide anion (0.15 mg/ml) scavenging abilities.

**Discussion:**

The results showed that ATPS was an efficient method to extract polysaccharides and could be used for the extraction of other polysaccharides. These results indicated that ALPs had great prospects as a functional food and could be exploited in multiple fields.

## Introduction

*Arctium lappa* L., also called “black radish” or “ginseng,”—is a straight-root biennial herb belonging to *Asteraceae* (Compositae) family (1, 2)—that was cultivated in Europe approximately 3,000 years earlier (3). Warm and humid climates are suitable for *Arctium lappa* L. It mainly grows in Northeast, North, and Northwest China (4). *Arctium lappa* L. root is a very popular vegetable in China, which is rich in the soluble dietary fiber found in inulin, polysaccharides, polyphenols, flavonoids, arctiin, and other active ingredients (5, 6), used as a kind of nutritive and functional food in traditional Chinese medicine (TCM), has very high nutritional and medicinal value, such as antioxidant, hypoglycemic, and lipid-lowering effects; immunity enhancement; bacteriostasis; and anti-inflammatory and other effects (7–9).

Polysaccharide is a kind of polymeric polymer compound with more than 10 monosaccharides linked by glycosidic bonds (10, 11). Studies have shown that polysaccharides have a variety of biological activities, including improving immunity, inhibiting the proliferation and diffusion of tumor cells, and regulating blood sugar balance (12–14). As a natural metabolic regulatory factor, polysaccharides have broad development prospects. With the continuous development of the social economy, the enormous potential value of ALPs is gradually recognized by people, and its functional food is increasingly favored by consumers (15). Currently, research on ALPs focuses on processing, physiological functions, and their applications in food. However, most of the ALP extraction methods are traditional and inefficient. Furthermore, the traditional extraction methods of ALPs are time-consuming and inefficient. There have been few studies on the antioxidant activity of ALPs (16, 17). Meanwhile, the biological activity of polysaccharides is related to the composition and structure of polysaccharides; therefore, it is essential to analyze the monosaccharide composition and structure of ALPs (18, 19).

Currently, the hot water extraction method, enzyme-assisted extraction, the lye extraction method, the ultrasonic-assisted method, and the microwave-assisted method are frequently used to extract plant polysaccharides (16, 20–22). Aqueous two-phase system (ATPS) is a new extraction technology developed in recent years, which has the advantages of simple operation, mild conditions, and environmental protection (23). When the nature of the extraction system is different, the substance enters the double aqueous phase system. Due to the surface properties, the charge effect, various forces (water-repellent bonding, hydrogen bonding, ionic bonding, etc.), and the influence of environmental factors, their concentration in the upper and lower phases are different. To improve the extraction rate and efficiency of ALPs, PEG-(NH_4_)_2_SO_4_, the ATPS method was used to extract ALPs from *Arctium lappa* L. root in the present study, and FTIR, SEM, and HPLC were used to determine the structure and composition of ALPs. In addition, the antioxidant abilities of ALPs were investigated to lay the foundation and provide the basis for further functional food development and application.

## Materials and methods

### Materials and reagents

The *Arctium lappa* L. root was purchased from the local market of Lanzhou, Gansu Province, China. Dry *Arctium lappa* L. root was crushed by a BJ-400 high disintegrator (Yongkang Boou Instrument Co., Ltd., Zhejiang, China) and treated with a sieving of 80 meshes. The screen underflow was degreased with normal hexane (5:1, v/m) for 12 h. The degreased powder was dried using a DHG-9030A oven (Shanghai Grows Instrument Co., Ltd., China).

Monosaccharide standard products, acetonitrile, 1,1-diphenyl-2-trinitrophenylhydrazine (DPPH), and trifluoroacetic acid (TFA) were purchased from Sigma–Aldrich Chemical Co., Ltd (St. Louis, USA). Sulfuric acid and ammonium sulfate were purchased from Sinopharm Chemical Reagent Co., Ltd (Beijing, China), and all other reagents used in this study were of analytical grade.

### Extraction of ALPs

#### Preparation of crude ALPs

An amount of 5 g degreased *Arctium lappa* L. powder was added into 150 ml distilled water with a ratio of material to liquid at 1:30; then the mixture was incubated at 50°C for 2.0 h, centrifuged (5,000 rpm, 20 min) to obtain the supernatant by Heraeus Multifuge X1R (Thermo Co., America). Sevag reagent (n-butanol: chloroform, 1:5, v/v) was added into the supernatant to remove proteins and the solution was centrifuged (5,000 rpm, 20 min) to obtain the crude ALPs.

#### Preparation of PEG-(NH_4_)_2_SO_4_ with ATPS

The ATPS studied in this article constituted PEG solution, (NH_4_)_2_SO_4_ solution, crude ALP solution, and deionized water, and the total quality of the ATPS was set as 20 g. A certain amount of PEG, ALP extract, and ammonium sulfate was added to the test tube, mixed and dissolved, and filled up to 20 ml with distilled water. The mixture was shaken for 2 min and extracted by a water bath at a certain temperature for 30 min. The solution was centrifuged at 4,000 rpm for 5 min to form two stable phases. The volume and absorbance of the upper and lower phases were measured, respectively. For details, refer to the study by Zhang et al. (24).

#### Determination of the extraction rate of ALPs

The content of ALPs was determined by the phenol–sulfuric acid method (25). A volume of 0.1 g/ml glucose standard solution at different concentrations (0.2, 0.4, 0.6, 0.8, and 1.0 ml) was removed with 1.0 ml of 6% phenol and added into different test tubes. Then, 5.0 ml of concentrated sulfuric acid was immediately added, shaken well, and allowed to stand for 30 min. The absorbance of samples was measured at 490 nm by an ultraviolet-visible spectrophotometer (Shanghai Spectrum Instrument Co., Ltd., Shanghai, China). A volume of 2.0 ml of distilled water was used as blank control. Using the different concentrations of the D-glucose (*x*) as the abscissa and the absorbance value (*y*) as the ordinate, a standard curve was drawn, and the linear regression equation (*y* = 103.161*x* – 2.3722, *R*^2^ = 0.9996) was obtained for calculating the sample concentration according to the absorbance value of the sample.

The ALP contents were calculated from the glucose standard curve and the ALP extraction rate was calculated according to the following equations:

(1) $$\begin{array}{l} Y=\frac{C_{b}V_{b}}{\left(C_{b}V_{b}+C_{t}V_{t}\right)}\times 100\% \end{array}$$

(2) $$\begin{array}{l} K=\frac{C_{t}}{C_{b}} \end{array}$$

(3) $$\begin{array}{l} R=\frac{V_{t}}{V_{b}}, \end{array}$$

where *y* is the extraction rate of ALPs of the lower phase (%), *K* is the distribution coefficient of polysaccharide in the ATPS, *R* is the volume ratio of the upper and lower phases, *V*_t_ is the upper phase volume (ml), *V*_b_ is the lower phase volume (ml), *C*_t_ is the mass concentration of polysaccharide in the upper phase (mg/ml), and *C*_b_ is the mass concentration of polysaccharide in the lower phase (mg/ml).

#### Experimental design of optimization extraction conditions

In the single-factor experiments, four variables, including the PEG molecular weights (1,000, 2,000, 4,000, 6,000, and 8,000), the quality fractions of PEG (10, 15, 20, 25, and 30%), the quality fractions of (NH_4_)_2_SO_4_ (10, 14, 18, 22, and 26%), and the extraction temperatures (50, 60, 70, 80, and 90°C) were taken into consideration to investigate their effects on the extraction rate of ATPS.

According to the results of single-factor tests, the optimal extraction parameters of ATPs were performed by response surface methodology (RSM) with a Box–Behnken design (BBD) to obtain the maximum extraction rate of ATPs. The quality fraction of PEG (X_1_, %), the quality fraction of (NH_4_)_2_SO_4_ (X_2_, %), and extraction temperature (X_3_, °C) were used as individual variables, and the ALPs extraction rate (Y) was used as the response value. A total of 17 experiments were conducted on a 3-factor, 3-level BBD with 5 replicates of central and 12 of factorial points (−1, 0, and 1). Test factors and horizontal design are shown in Table 1. The experimental data were fitted to a second-order polynomial regression model using the following equation:

(4) $$\begin{array}{l} \text{Y}={\text{B}}_{0}+\overset{\text{n}=3}{\underset{\text{i}=1}{\sum }}{\text{B}}_{\text{i}}{\text{X}}_{\text{i}}+\overset{\text{n}=3}{\underset{\text{i}=1}{\sum }}{\text{B}}_{\text{ii}}{\text{X}}_{\text{i}}^{2}+\overset{\text{n}=3}{\underset{\text{i}=1}{\sum }}{\text{B}}_{\text{ij}}{\text{X}}_{\text{i}}\text{Yj} \end{array}$$

where *Y* is the response variable (ALPs extraction rate, %); *B*_0_, *B*_*i*_, *B*_*ii*_, and *B*_*ij*_ are the regression coefficients of variables for the intercept, the linear term, the quadratic term, and the interaction term, respectively; and *X*_*i*_ and *X*_*j*_ are the independent variables (*i* ≠ *j*).

**Table 1:** The process parameters setting for ALPs extraction, according to Box–Benkhen design.

**Factor**	**Coded factor levels**		
	−**1**	**0**	**1**
X_1_-PEG quality fraction/%	20	25	30
X_2_-(NH_4_)_2_SO_4_ quality fraction/%	14	18	22
X_3_-extraction temperature/°C	60	70	80

#### FT-IR analysis of ALPs

We mixed and ground 1 mg ALPs with 500 mg KBr, and the mixed samples were pressed for 5 min using an HYP-15 machine (Tianjin Port East Technology Co., Ltd, China). The chemical bonds and functional groups of polysaccharides were analyzed by scanning in the mid-infrared frequency range of 4,000–400 cm^−1^ using FTIR spectrometer (FTIR-650, Tianjin Guangdong Science and Technology Co., Ltd, China) (26).

#### SEM analysis of ALPs

The ALPs were fixed on the specimen stage with an electrically conducting adhesive, and the samples were coated with gold sputtering. Then, the surface morphology of the samples was observed using a ZEISS EVO18 scanning electron microscope (Carl Zeiss AG, Bruker Co., Germany) at an accelerating voltage of 10 kV with different magnifications (27).

#### Monosaccharide composition analysis of ALPs

The monosaccharide components of the ALPs were measured by the method of Chen et al. (28) with slight modifications. Briefly, 10.00 mg of the crude ALP sample was precisely weighed and put into a 10-ml test tube, and 5 ml of the TFA solution (2 mol/L) was added. Then, the sample was hydrolyzed in a water bath (100°C, 5 h). After cooling, the pH value of the mixture solution was adjusted to 7.0 by adding 3 mol/L NaOH and centrifuged (5,000 rpm, 10 min) to obtain the supernatant. Volumes of 0.2 ml PMP methanol solution (0.5 mol/L) and 0.2 ml NaOH (0.3 mol/L) were added into the ALP supernatant after hydrolysis and mixed well. It was incubated in a hot water bath at 70°C for 1 h and then 0.2 ml of 0.3 mol/L HCl solution and 1 ml chloroform were added to the supernatant, and the upper aqueous phase was filtered through a membrane of 0.22 μm to produce the solution to be tested. Rhamnose, glucose, galactose, fructose, and arabinose standards were treated as same as the sample.

A 1260 Infinity HPLC System (Agilent, USA) coupled with a diode array detector (DAD) was used to detect the monosaccharide components of ALPs, HPLC conditions were set as follows: a mobile phase of 0.02 mol/L phosphate buffer (pH 6.8) and acetonitrile in a ratio of 81:19 (v/v), an Agilent ZORBAX Eclipse XDB-C18 column (4.6 mm × 250 mm, 5 μm, Agilent Co., USA), a column temperature of 28°C, a flow rate of 1 ml/min, sample volume of 5 μl, and a detection wavelength of 250 nm.

### *In vitro* antioxidant activity assay

#### ·OH radical scavenging activity

The OH scavenging activity of the ALPs was determined according to the Fenton-type reaction studied by Wang et al. (29). We added 1 ml of ALPs solution at different concentrations (0.2, 0.4, 0.6, 0.8, and 1.0 mg/ml), 1 ml FeSO_4_ solution (9 mmol/L), 1 ml salicylic acid solution (9 mmol/L), and 1 ml H_2_O_2_ solution (8.8 mmol/L) into a 10-ml test tube and reacted in a water bath at 37°C for 30 min. The absorbance values were measured at the wavelength of 510 nm. V_C_ solution with different concentrations (0.2, 0.4, 0.6, 0.8, and 1.0 mg/ml) was used as a positive control. All experiments were carried out in three parallel samples, and the average value was used to calculate the OH scavenging activity of ALPs. The scavenging rate was calculated according to the following equation:

(5) $$\begin{array}{l} \text{E}=\left(1-\frac{{\text{A}}_{1}-{\text{A}}_{2}}{{\text{A}}_{0}}\right)\times 100\text{\%} \end{array}$$

where E is the scavenging activity of the ·OH radical (%); A_0_ is the absorbance value of blank control; A_1_ is the absorbance value of the samples; and A_2_ is the absorbance value of the sample without the ·OH radical.

#### DPPH radical scavenging activity

The DPPH radical scavenging activity of ALPs was measured by the method of Liu et al. (30). A volume of 2 ml DPPH solution (0.2 mmol/L) was mixed with 2 ml ALPs samples at different concentrations (0.2, 0.4, 0.6, 0.8, and 1.0 mg/ml) and placed in the unlit environment at room temperature for 30 min. We used 2.0 ml of distilled water instead of ALPs as control 1, 2.0 ml of sample solution and 2.0 ml of distilled water as control 2, and 2.0 ml DPPH solution and 2.0 ml V_C_ solutions of 0.2, 0.4, 0.6, 0.8, and 1.0 mg/ml were used as positive control. The absorbance values of each experimental group were measured at 517 nm. The absorbance values were substituted into the following equation to calculate the scavenging percentage of DPPH radical:

(6) $$\begin{array}{l} \text{E}=\left(1-\frac{{\text{A}}_{\text{a}}-{\text{A}}_{\text{c}}}{{\text{A}}_{\text{b}}}\right)\times 100\text{\%} \end{array}$$

where E is DPPH radical scavenging activity (%); A_a_ is the absorbance values of the samples; and A_b_ and A_c_ are the absorbance values of control 1 and control 2, respectively.

#### ·${\text{O}}_{2}^{-}$ radical scavenging activity

The method of Liu et al. was used to measure the ${\text{O}}_{2}^{-}$ scavenging activity of the ALPs (31). We mixed 1 ml ALPs solution with different concentrations (0.2, 0.4, 0.6, 0.8, and 1.0 mg/ml) and 4.5 ml Tris-HCl solution (1 mol/L, pH 8.2) and reacted at 50°C for 30 min. Then, 1 ml of pyrogallic acid (2 mmol/L) was added and placed in a 20°C water bath for 10 min. Finally, two drops of HCl solution (8 mol/L) were immediately added to terminate the reaction; 0.3 ml distilled water was used to substitute pyrogallol instead of pyrogallic acid; and the V_C_ solution at different concentrations (0.2, 0.4, 0.6, 0.8, and 1.0 mg/ml) was used as positive control instead of the ALP solutions. The absorbance was measured at 320 nm and the ${\text{O}}_{2}^{-}$ scavenging activity was calculated by substituting into the following equation:

(7) $$\begin{array}{l} \text{E}=\frac{{\text{A}}_{\text{ii}}-{\text{A}}_{\text{i}}}{{\text{A}}_{\text{ii}}}\times 100\text{\%} \end{array}$$

where *E* is the scavenging activity of the ·${\text{O}}_{2}^{-}$ radical (%) and *A*_*i*_ and *A*_*ii*_ were the absorbance values of the control and samples, respectively.

### Statistical analysis

All experimental data were analyzed by the ORIGIN 2018 Graphing and Analysis and SPSS 25.0 software (IBM, SPSS, Chicago, USA), and the data results were expressed as mean value ± standard deviation (SD). Response surface experimental data were statistically analyzed using the Stat-Ease 360 version 8.0.6 software (Stat-Ease, Inc., Minneapolis, USA). Significance among different groups was analyzed by a one-way analysis of variance (ANOVA), followed by Duncan's test, and all experiments or analyses were performed in triplicate.

## Results and discussion

### Effects of PEG molecular weight, PEG quality fraction, (NH_4_)_2_SO_4_ quality fraction, and extraction temperature on the extraction rate of ALPs

**Figure 1:**
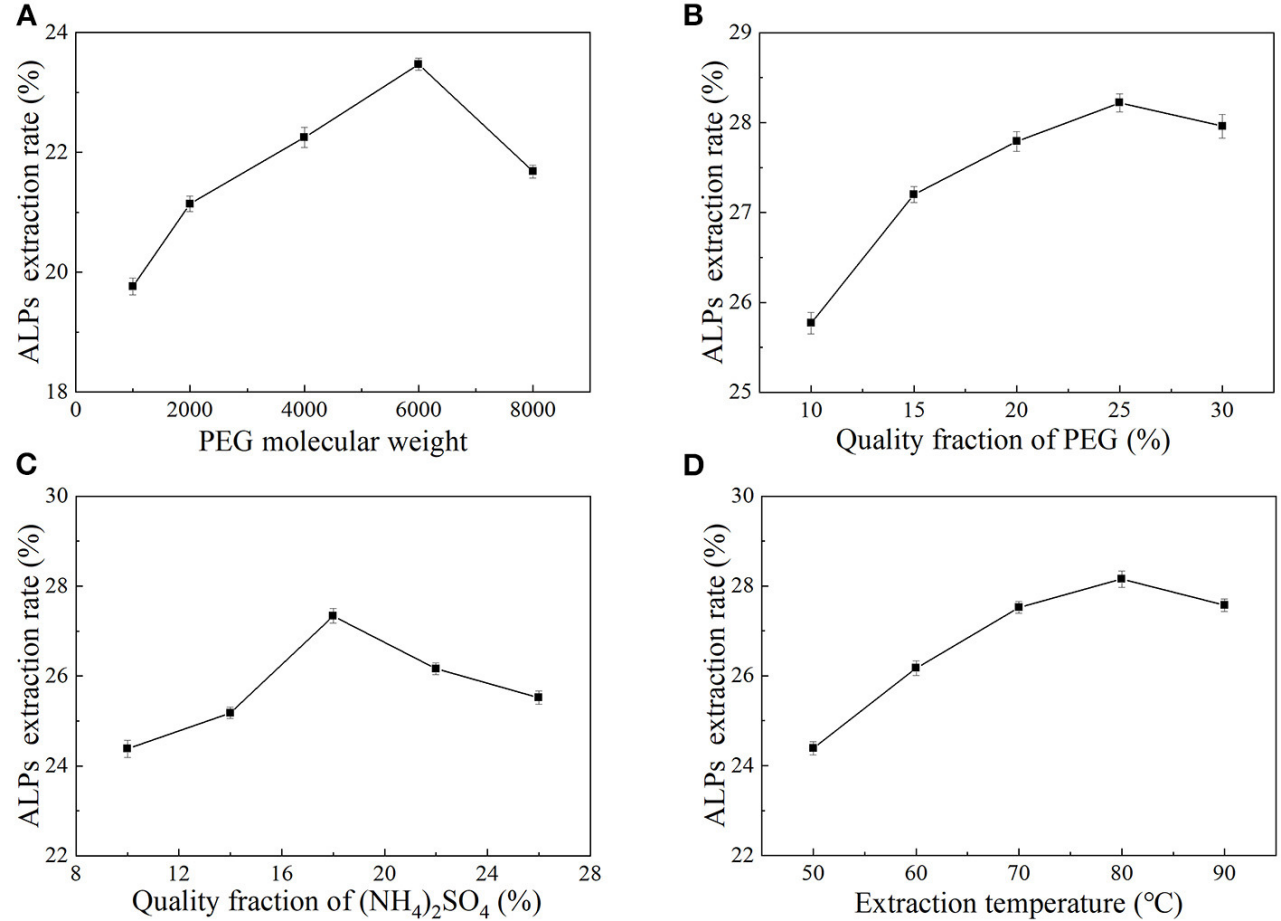
The effects of PEG molecular weight **(A)**, PEG quality fraction **(B)**, (NH_4_)_2_SO_4_ quality fraction **(C)**, and extraction temperature **(D)** on the extraction rate of ALPs.

Due to its good solubility in water and many organic solvents and its non-toxicity, PEG is considered a kind of non-ionic polymer. PEG has been widely used to extract polysaccharides from a variety of plants (32). As shown in Figure 1A, when the PEG molecular weight was between 2,000 and 6,000, the extraction rate of ALPs increased significantly. When the PEG molecular weight was 6,000, the extraction rate of ALPs reached a maximum of 23.47 ± 0.08%. However, when the PEG molecular weight exceeded 6,000, the extraction rate of ALPs decreased gradually. The molecular weight and hydrophobicity of PEG affected the extraction capacity of the two-phase aqueous extraction system (33). As the molecular weight of PEG increased, the molecular chain became longer, the relative fraction of hydroxyl decreased, and the higher molecular weight grades were less hydrophilic and had stronger hydrophobicity (34, 35). Therefore, to accumulate the extracted substance as much as possible in the upper phase, PEG of lower molecular weight was selected and *vice versa*. Therefore, the optimum PEG molecular weight was 6,000. At the same time, PEG-6000 was also selected as the extraction solvent for the extraction of aloe polysaccharide by Xing et al. (36).

Figure 1B shows the influence of PEG quality fraction on the extraction rate of ALPs. In the range of 10%−30%, the extraction rate of ALPs increased first and then decreased with the increase of PEG quality fraction, and the ALP extraction rate reached the maximum value (28.22 ± 0.09%) when a quality fraction of PEG was 25%. The main reason for the increase in the extraction rate of ALPs may be that, when the quality fraction of PEG increased, water molecules in the system may have gathered in the lower phase, and the volume of the lower phase increased, dissolving more ALPs (37). The high PEG quality fraction leads to a high viscosity solution, which hinders the transfer of polysaccharide molecules and causes the ALPs in the upper phase to remain between the two phases (38). Considering all aspects, the quality fraction of PEG of 25% was selected as the optimal PEG quality fraction, and it was chosen as the central point of RSM.

Inorganic salts can ionize positive or negative ions in solution, and the tendency of liquid-liquid phase separation is related to the interaction between inorganic salt ions and water molecules, which affects the distribution of extracted substances (39–41). The influence of (NH_4_)_2_SO_4_ quality fraction on the extraction rate of ALPs is shown in Figure 1C. With the increase of an (NH_4_)_2_SO_4_ quality fraction, the extraction rate of ALPs increased significantly as the (NH_4_)_2_SO_4_ quality fraction increased from 10% to 18% and then decreased when the (NH_4_)_2_SO_4_ quality fraction increased from 18% to 26%. When the (NH_4_)_2_SO_4_ quality fraction reached 18%, the extraction rate of ALPs reached the maximum value at 27.34 ± 0.12%. The main reason for this phenomenon may be due to the increase of an (NH_4_)_2_SO_4_ quality fraction, the improvement in the separation performance of the two aqueous phases and the increase in the volume of the lower phase of the system, which is beneficial to the aggregation of polysaccharides in the lower phase. However, when the (NH_4_)_2_SO_4_ quality fraction was too high, the “salting out” effect was enhanced in the presence of (NH_4_)_2_SO_4_, and the solubility of polysaccharides was reduced (42), which is the main reason for the decrease in the extraction rate of ALPs. Therefore, the (NH_4_)_2_SO_4_ quality fraction of 18% as the center point of RSM and the optimal (NH_4_)_2_SO_4_ quality fraction were selected.

According to the graph of temperature vs. ALPs extraction rate (Figure 1D), it was observed that the extraction rate of ALPs increased significantly when the extraction temperature was in the range of 50°C−80°C and rose to the maximum value (25.75 ± 0.10%) at 80°C. However, when the temperature exceeded 80°C, the extraction rate of ALPs decreased. This may be because that higher temperature could enhance the solubility and diffusion coefficient of ALPs in the extracting solvent, which could improve the extraction rate of ALPs. However, when the extraction temperature was too high, the volume of the upper and lower phases changed, the energy consumption increased, and the amount of polysaccharide dissolution increased, leading to a decrease in the ALP extraction rate. Therefore, the extraction temperature should not be too high in work. With careful consideration, the extraction temperature of 80°C was selected as the center point of RSM.

### Analysis of the response surface

#### Statistical analysis and the model fitting

The results of the three-factor three-level experimental data are shown in Table 2. The *Y* (ALP extraction rate) range was 23.55%−28.96%. A total of 17 group experiments were designed by the Design-Expert 8.0.6 software; after multiple regression fitting, the second-order polynomial equation of the response regression model was obtained, as shown in the following equation:

(8) $$\begin{array}{l} \text{Y}=28.83+0.71X_{1}+1.21X_{2}+0.18X_{3}+0.26X_{1}X_{2}\\ \;-0.14X_{1}X_{3}-0.25X_{2}X_{3}-0.63X_{1}^{2}\\ \;-2.01X_{2}^{2}-1.62X_{3}^{2}, \end{array}$$

where *Y* is the ALPs rate (%) and *X*_1_, *X*_2_, and *X*_3_ are the coded values of the PEG quality fraction (%), (NH_4_)_2_SO_4_ quality fraction (%), and extraction temperature (°C), respectively.

**Table 2:** Box–Benkhen design of the independent variables and experimental values of ALPs rate.

**No**.	**Factor**			**Y (ALPs extraction rate)/%**
	**X**_1_ **-PEG quality fraction/%**	**X**_2_**-(NH**_4_**)**_2_**SO**_4_ **quality fraction/%**	**X**_3_**-extraction temperature/** °**C**	
1	−1	−1	0	24.65 ± 0.003
2	1	−1	0	25.39 ± 0.016
3	−1	1	0	26.49 ± 0.016
4	1	1	0	28.27 ± 0.003
5	−1	0	−1	25.47 ± 0.063
6	1	0	−1	27.32 ± 0.040
7	−1	0	1	26.14 ± 0.040
8	1	0	1	27.42 ± 0.063
9	0	−1	−1	23.55 ± 0.056
10	0	1	−1	26.53 ± 0.046
11	0	−1	1	24.39 ± 0.046
12	0	1	1	26.37 ± 0.056
13	0	0	0	28.75 ± 0.160
14	0	0	0	28.94 ± 0.013
15	0	0	0	28.81 ± 0.003
16	0	0	0	28.71 ± 0.010
17	0	0	0	28.96 ± 0.005

An analysis of variance (ANOVA) was performed on the experimental data to assess the significance and applicability of the quadratic model. The significance of each coefficient was estimated by comparing the magnitude of the *p*-value. The smaller the *p*-value, the more significant the coefficient, and vice versa (43). The results of ANOVA analysis are presented in Table 3. It was found that the *p*-value of the response regression model had a very high significance (*p* < 0.0001), indicating that the model had good applicability to this experiment. The lack-of-fit *F*-value of 1.46 and the lack-of-fit *p*-value of 0.3525 implied that the lack of fit was not significant relative to the pure error. The determination coefficient (*R*^2^) of the model was 0.9951, which indicated that this predicted model can explain 99.79% of the results, and only 0.49% of the total variance was not explained by the model. The adjustment determination coefficient ($R_{\text{Adj}}^{2}$) was 0.9951, indicating there was a good agreement between the experimental values and predicted values.

**Table 3:** Results of variance analysis of quadratic multinomial simulation.

**Source**	**Sum of square**	**DF**	**Mean square**	***F* value**	***P* value**	**Significance**
Model	48.97	9	5.44	363.26	< 0.0001	**
X_1_	3.99	1	3.99	266.41	< 0.0001	**
X_2_	11.71	1	11.71	782.01	< 0.0001	**
X_3_	0.26	1	0.26	17.55	0.0041	**
X_1_ X_2_	0.27	1	0.27	18.05	0.0038	**
X_1_ X_3_	0.081	1	0.081	5.42	0.0527	
X_2_ X_3_	0.25	1	0.25	16.69	0.0047	**
${\text{X}}_{1}^{2}$	1.66	1	1.66	110.96	< 0.0001	**
${\text{X}}_{2}^{2}$	16.94	1	16.94	1130.94	< 0.0001	**
${\text{X}}_{3}^{2}$	11.03	1	11.03	736.17	< 0.0001	**
Lack of fit	0.055	3	0.018	1.46	0.3525	
Pure error	0.050	4	0.013			
Cor total	49.07	16		R^2^ = 0.9979 ${\text{R}}_{\text{Adj}}^{2}$ = 0.9951		

^*^Significant at the 5% level (*p* < 0.05); ^**^Significant at the 1% level (*p* < 0.01).

Table 3 shows the analysis of the significance detection results of each factor. It was clear from the table that the *p*-value of the first terms *X*_1_ (*p* < 0.0001), *X*_2_ (*p* < 0.0001), and X_3_ (*p* = 0.0041) were lower than 0.01, which indicated that the PEG quality fraction (*X*_1_), (NH_4_)_2_SO_4_ quality fraction (*X*_2_), and extraction temperature (*X*_3_) showed a significant impact on the extraction rate of ALPs. It was easy to determine that the PEG quality fraction and the (NH_4_)_2_SO_4_ quality fraction had a more significant impact on the extraction rate of ALPs than the extraction temperature. The second terms $X_{1}^{2}$ (*p* < 0.0001), $X_{2}^{2}$ (*p* < 0.0001), and $X_{3}^{2}$ (*p* < 0.0001) were also highly significant at the 1% level. The *p*-values of interaction items *X*_1_*X*_2_ (*p* = 0.0038) and *X*_2_*X*_3_ (*p* = 0.0047) were lower than 0.01, which was highly significant at the 1% level, and the interaction item *X*_1_*X*_3_ (*p* = 0.0527 > 0.05) was not significant. These *p*-values of interaction item indicated intense interaction between a PEG quality fraction and the (NH_4_)_2_SO_4_ quality fraction, an (NH_4_)_2_SO_4_ quality fraction and extraction temperature, and the interaction between a PEG quality fraction and extraction temperature were weaker.

#### Response surface plot and contour plot analyses

**Figure 2:**
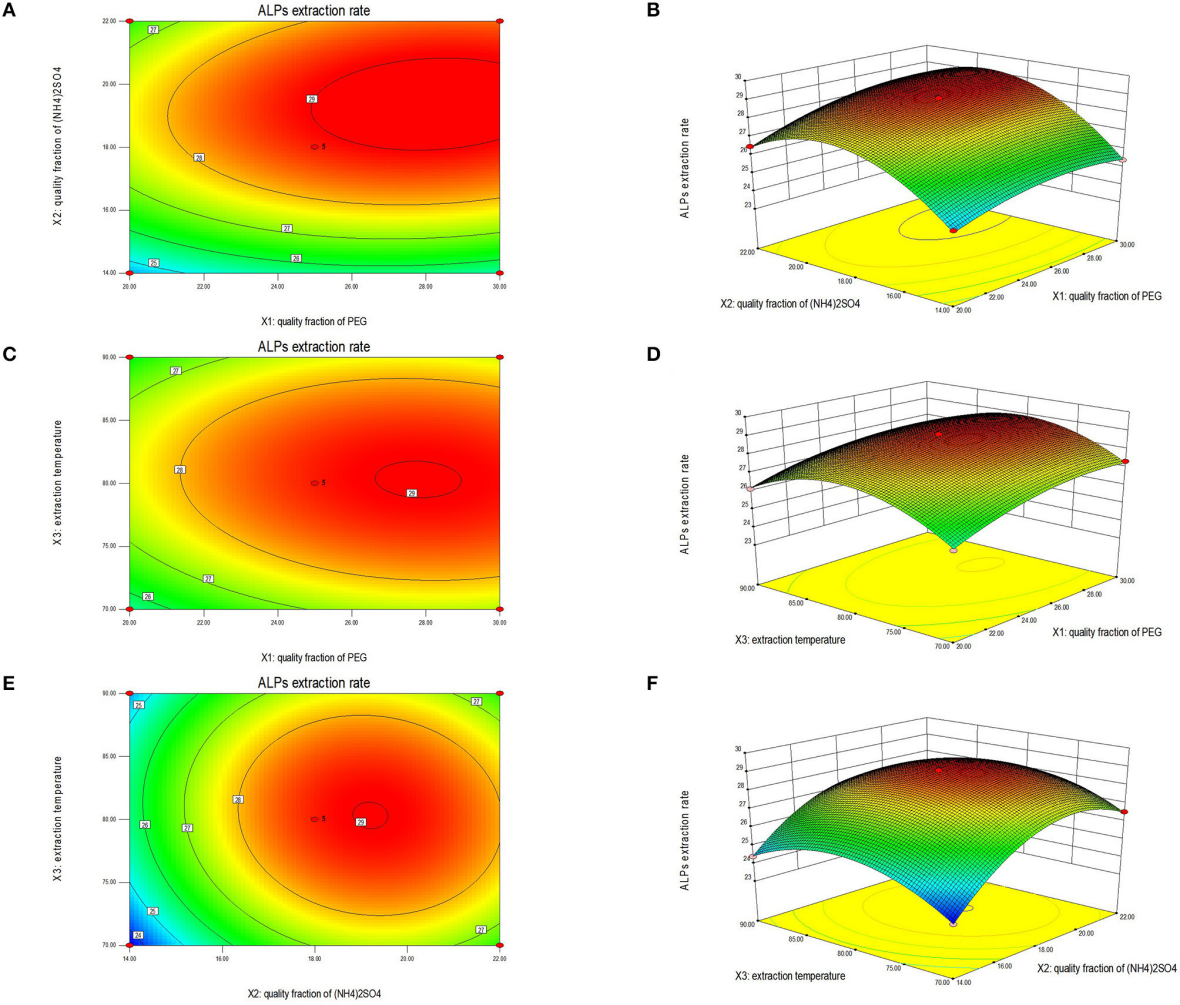
The 2D contour plot and 3D response surface showing the effect of PEG quality fraction and (NH_4_)_2_SO_4_ quality fraction **(A, B)**, PEG quality fraction and extraction temperature **(C, D)**, (NH_4_)_2_SO_4_ quality fraction and extraction temperature **(E, F)** on the extraction rate of ALPs.

Studies have shown that the shape of the contour and response surface can reflect the strength of the interaction between the factors. The closer the contour was to an ellipse, the more vital the interaction between these two factors. If the interaction between the two factors was weak, the contour line was closer to a circle (31, 44). There was strong interaction among the factors, and if the surface diagram was steeper, the influence was more significant (43). The surface diagram of ALPs extraction results between any two factors was analyzed systematically to determine the optimal factor level and evaluate the effect on the extraction rate of ALPs. According to the regression equation, the 2D contour plots and 3D response surfaces generated are displayed in Figure 2. Their significance is consistent with the results of ANOVA.

Figures 2A, B show the 2D contour plot and 3D response surface of the interaction effect of the PEG quality fraction and the (NH_4_)_2_SO_4_ quality fraction on the extraction rate of ALPs. If the contour line (Figure 2A) of the PEG quality fraction and the (NH_4_)_2_SO_4_ quality fraction was an ellipse, and the response surface (Figure 2B) was steep, the quality fraction of PEG and the (NH_4_)_2_SO_4_ quality fraction had a significant interaction on the extraction rate of ALPs. Moreover, when the ratio of the PEG quality fraction was 25%, and the (NH_4_)_2_SO_4_ quality fraction was 18%, the extraction rate of ALPs could reach the maximum value. Figures 2C, D show the interaction effects of a PEG quality fraction and extraction temperature on the extraction rate of ALPs. According to the contour line shown in Figure 2C, the ellipticity was insignificant; therefore, the interaction was considered weaker. It can be observed from Figure 2D that the 3D graphics were relatively flat, indicating that the interaction between the PEG quality fraction and extraction temperature was not significant. When the ratio of the PEG quality fraction was 25%, and the extraction temperature was 80°C, the extraction rate of ALPs could reach the maximum value. Figures 2E, F show the contour plots and response surfaces of (NH_4_)_2_SO_4_ quality fraction and PEG quality fraction on the extraction rate of ALPs. While the contour shape was elliptical as shown in Figure 2E, the 3D graphics is steep as shown in Figure 2F. It was clear from this result that there was a strong interaction between an (NH_4_)_2_SO_4_ quality fraction and extraction temperature. When the (NH_4_)_2_SO_4_ quality fraction was 18%, and the extraction temperature was 80°C, the extraction rate of ALPs reached maximum value.

#### Validation of the predictive model

The predicted extraction rate of ALPs was obtained under the following conditions: the quality fraction of PEG-6000 27.65%, the quality fraction of (NH_4_)_2_SO_4_ 19.18%, and the extraction temperature of 88.09°C. For operational convenience, the actual operating process conditions differed from the predicted optimal process conditions: the quality fraction of PEG-6000 was modified to 25%, the quality fraction of (NH_4_)_2_SO_4_ was 18%, and the extraction temperature was 80°C. The actual extraction rate of ALPs was 28.83%. The extraction rate was higher than the one discussed in the previous report by Guo et al. who reported that ALPs were extracted by ultrasound-assisted extraction method and the extraction rate was reached at 14.22% under solid–liquid radio of 1:20, with an extraction temperature of 55°C, extraction time of 55 min, and power of 175 W (45). Li et al. used a water extraction method to extract ALP, and the extraction rate was only 4.69% (16). It was indicated that the ATPS method had a better effect in extracting ALPs than the water extraction method and the ultrasonic-assisted method.

#### FTIR spectrum of ALPs

**Figure 3:**
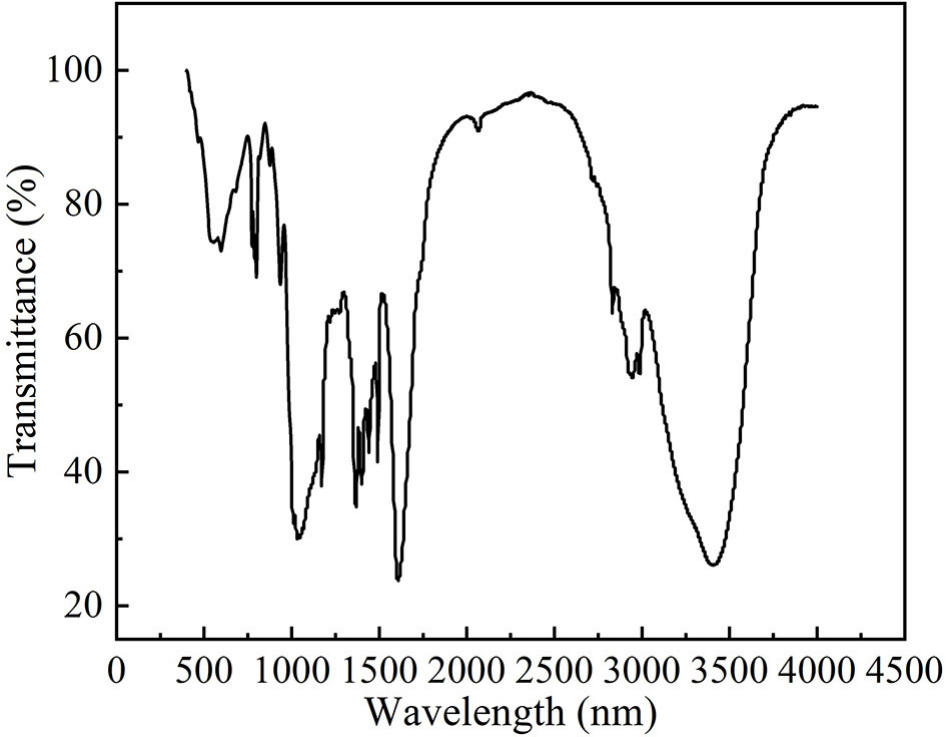
Infrared spectra of ALPs.

From the FTIR spectra of ALPs (Figure 3), we observed that the broad absorption peak at 3,500–3,200 cm^−1^ was due to the O–H stretching vibration and the peak at 2,950–2,900 cm^−1^ was due to C–H stretching vibration. The characteristic absorption peak of the acetyl group in the amide group at 1,620.5 cm^−1^ indicated the presence of aminosaccharide in ALPs, and the peak width of this characteristic peak indicated the low content of aminosaccharide. The peak at 1,348.1 cm^−1^ was a characteristic peak of the O–H bending vibration and at 1,283.7 cm^−1^ was a characteristic peak of the C–H bending vibration. The absorption peaks at 1,100–900 cm^−1^ indicated that ALPs had pyranose rings. The absorption peaks caused by the furan ring's C–H bending vibration and the pyran ring's C–O–C vibration were at 802.5 and 750.2 cm^−1^, respectively. The results indicate that ALPs are typical acidic polysaccharides.

#### Microstructure of ALPs

**Figure 4:**
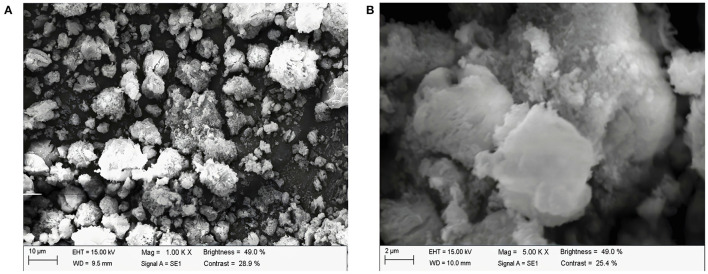
Scanning electron microscopy of ALPs [**(A)** 1,000×; **(B)** 5,000×].

Figures 4A, B show the images of ALPs at 1,000× and 5,000× magnifications under scanning electron microscopy, respectively. In addition, ALPs appear as clumps, with an uneven surface and an irregular shape at magnification of 1,000×. Moreover, ALPs consist of many small particles of irregular shape and size. The aggregation and adhesion of many molecules or molecular groups may cause it. When magnified to 5,000×, ALPs had a wooly, coarse, and fine surface. The results indicate that the attraction between polysaccharide molecules exists, and the repulsive force between molecules is relatively weak.

#### Monosaccharide composition of ALPs

**Figure 5:**
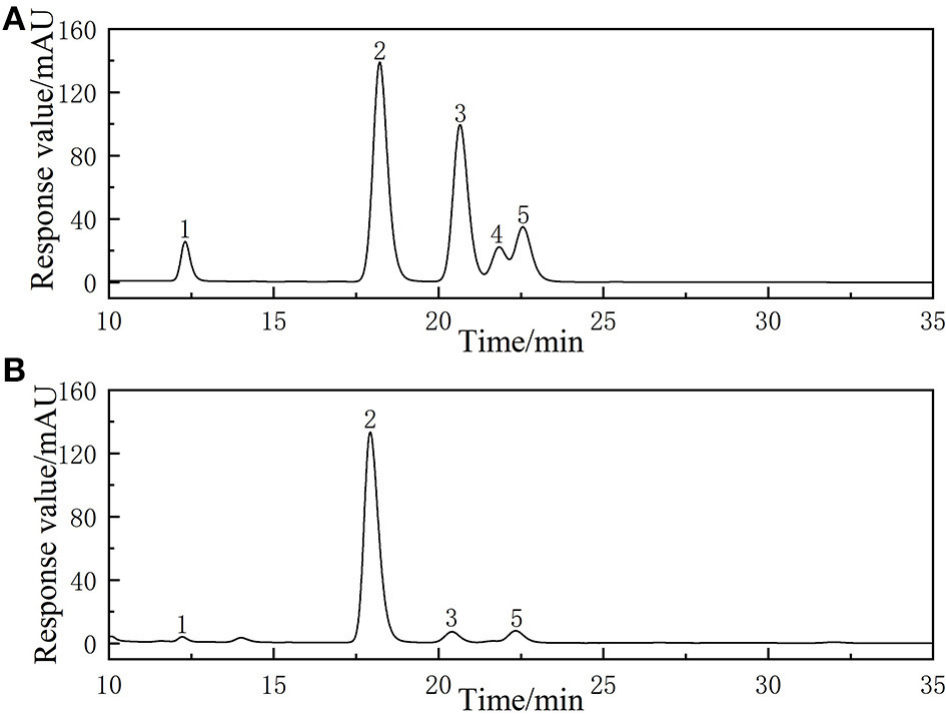
The HPLC chromatogram of **(A)** reference monosaccharides and **(B)** monosaccharides composition of ALPs.

The monosaccharide composition of ALPs was determined by HPLC and the results are shown in Figure 5. It was found that ALPs mainly consisted of glucose, rhamnose, arabinose, and galactose with a molar ratio of 70.19:10.95:11.16:6.90. Previous research reported by Juliane et al. found that the polysaccharide RF30 of a crude polysaccharide fraction [Soluble *Arctium lappa* L. polysaccharide (SAA)] from *Arctium lappa* L. leaves contained galacturonic acid, galactose, arabinose, rhamnose, and glucose (17). The results of Li et al. also proved that ALPs mainly contain arabinose and galactose (16). The research of Yu et al. found that the acid hydrolyzed fragment (ALP2-A) of pectin from *Arctium lappa* L. was composed of rhamnose and galacturonic acid, and enzymatically hydrolyzed fragment (ALP2-E) contained rhamnose, glucuronic acid, galacturonic acid, galactose, and arabinose (46).

### Antioxidant activity analysis

#### ·OH radical scavenging

**Figure 6:**
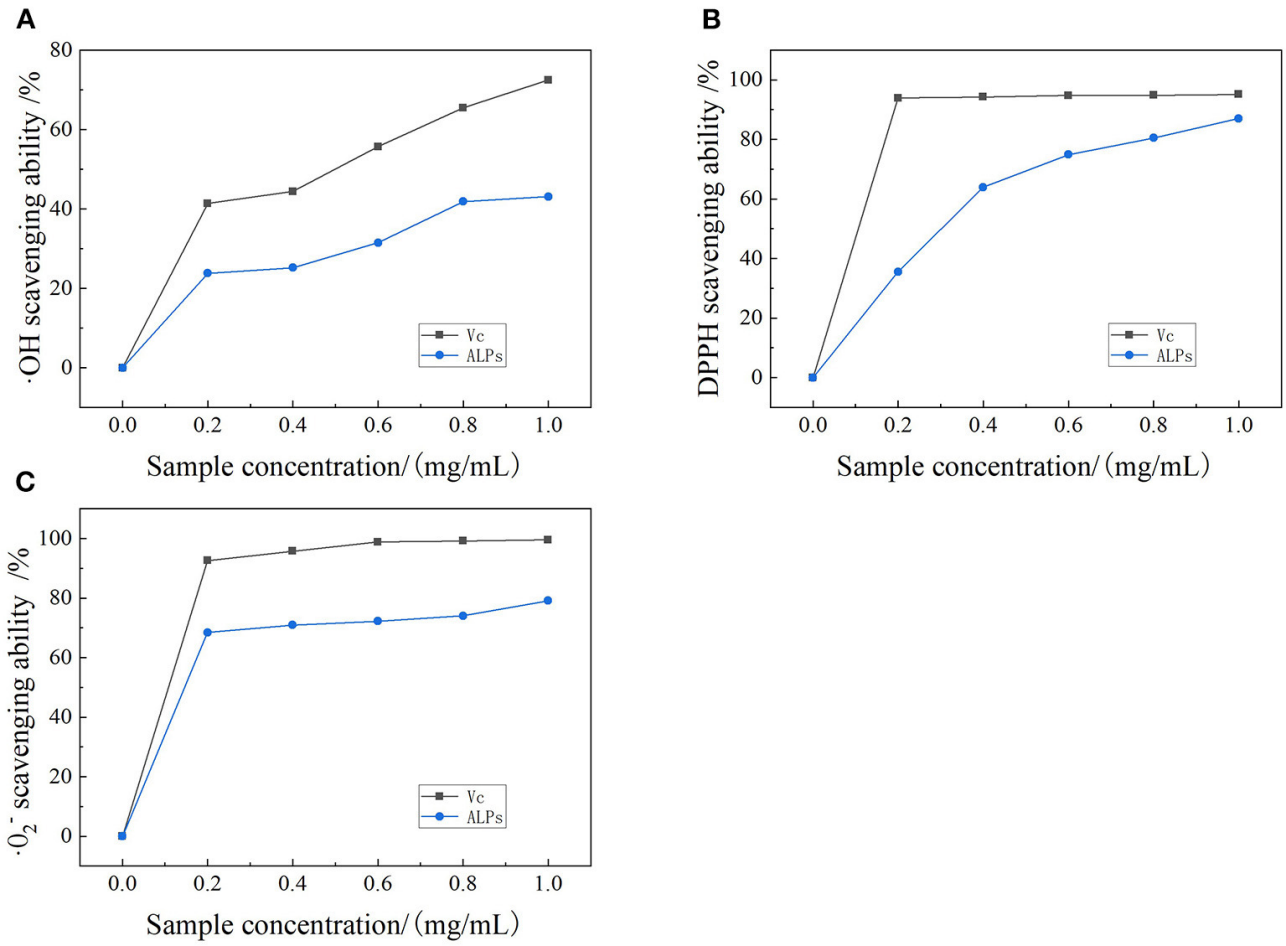
Scavenging effect of ALPs on **(A)** the ·OH radicals compared with V_C_, **(B)** the DPPH radicals compared with V_C_, and **(C)** the ·${\text{O}}_{2}^{-}$ radicals compared with V_C_.

It can be easily observed from Figure 6A that the OH radical scavenging capacity of ALPs is directly proportional to the concentration of ALPs. The scavenging ability of ALPs on the ·OH radical increased with increasing concentration of ALPs. It kept increasing until the OH radical scavenging rate was maximum (43.1%) at 1.0 mg/ml. The OH free radical can cause oxidative stress, triggering chronic diseases such as cancer and coronary heart disease (47). Furthermore, ALPs, which act as antioxidants, can regulate oxidative stress, reducing the potential harm of free radical oxidation (48). Therefore, hydroxyl radical scavenging ability is often used as one of the indicators to test antioxidant activity *in vitro* (49). The results indicated that ALPs had a certain degree of scavenging ability of the ·OH radical. In addition, the half-maximal inhibitory concentration (IC_50_) value of ALPs was 1.732 mg/ml, and the IC_50_ value of V_C_ was 0.385 mg/ml. The study by Liu et al. reported the OH radical scavenging capacity of *Arctium lappa* L. polysaccharides (ALP1) was only 4.20% at 0.3125 mg/ml, and the OH radical scavenging rate of ALP1 reached 99.19% at 2.5 mg/ml (7). It indicated that ALPs had a powerful ability to scavenge the ·OH radicals and can regulate oxidative stress, reducing the potential harm of free radical oxidation.

#### DPPH radical scavenging

Observing the DPPH radical scavenging capacity curve of ALPs (Figure 6B), it was found that the scavenging capacity of ALPs increased continuously in the range of 0.0–1.0 mg/ml, and the scavenging capacity of ALPs increased to the maximum value (87%). When the concentration of ALPs was 1.0 mg/ml, the DPPH radical scavenging capacity of ALPs was close to that of V_C_. DPPH radical is a stable free radical with a single electron, and its alcohol solution is purple (50, 51). Furthermore, ALPs, which act as antioxidants, can make the single electron pair and DPPH radical alcohol solution fade (52, 53). Hence, the strength of the antioxidant can be determined according to the absorption value of the solution (54). It can be observed that ALPs have concentration-dependent scavenging activity and scavenging solid ability on DPPH free radicals. Moreover, the IC_50_ of ALPs was 0.290 mg/ml, while the IC_50_ value of V_C_ was 0.11 mg/ml. A previous study reported that the mean IC_50_ value of DPPH radicals scavenging capacity of *A. lappa* root was 29.65 ± 4.03 μg/ml (55). Also, ALPs extracted in this study had stronger DPPH clearing ability.

#### Scavenging superoxide anion radicals

Superoxide anion (·${\text{O}}_{2}^{-}$) free radical is one of the free radicals with high activity and toxicity, leading to the formation of other toxic reactive oxygen species (ROS), which cause tissue damage and various diseases (56, 57). The ${\text{O}}_{2}^{-}$ scavenging ability of ALPs increased continuously within the test range from 0 mg/ml to 1.0 mg/ml (Figure 6C). The ·${\text{O}}_{2}^{-}$ scavenging ability of ALPs was increased rapidly and then slowly decreased to the range of 0.0–1.0 mg/ml. The ${\text{O}}_{2}^{-}$ scavenging rate of ALPs reached the maximum of 79.1% at 1.0 mg/ml. These results indicate that ALPs had a strong scavenging ability on the ·${\text{O}}_{2}^{-}$ free radical. Meanwhile, the IC_50_ value of ALPs and V_C_ were 0.15 and 0.10 mg/ml. When compared with the result of Skowrońska et al. who reported that the mean IC_50_ value of the ·${\text{O}}_{2}^{-}$ radical scavenging capacity of *A. lappa* root was 27.50 ± 8.20 μg/ml (55), ALPs had a more vital scavenging ability against the ·${\text{O}}_{2}^{-}$ radicals.

## Conclusion

*Arctium lappa* L. root is rich in polysaccharide, which has antioxidant, antitumor, and immune regulation effects, and has broad development prospects. In the present study, the ALPs were extracted by the ATPS method. The effects of PEG molecular weight, a quality fraction of PEG, a quality fraction of (NH_4_)_2_SO_4_, and the extraction temperature on the extraction rate of ALPs were studied, and RSM optimized the extraction process conditions of ALPs. The optimal extraction conditions were obtained as follows: the quality fraction of PEG-6000 was 25%, the quality fraction of (NH_4_)_2_SO_4_ was 18%, and the extraction temperature was 80°C. The extraction rate of ALPs could reach the maximum value of 28.83%. Both FTIR and SEM analyses of ALPs showed that ALPs were specific polysaccharides composed of irregular shapes and sizes of particles. The surface was uneven, and there was an attraction between the polysaccharide molecules. The results of HPLC showed that the ALPs were mainly composed of glucose, rhamnose, arabinose, and galactose with a molar ratio was 70.19:10.95:11.16:6.90. Finally, the result of the *in vitro* antioxidant experiment of ALPs showed that ALPs had a certain degree of scavenging ability on ·OH radical, DPPH radical, and ·${\text{O}}_{2}^{-}$ radical; their scavenging activities increased with the increase in polysaccharide concentration, and their IC_50_ values of ALPs were 1.732, 0.290, and 0.15 mg/ml, respectively. These results suggest that *Arctium lappa* L is a promising functional food.

## Data availability statement

The original contributions presented in the study are included in the article/supplementary material, further inquiries can be directed to the corresponding author.

## Author contributions

JY and DG took the initiative for this work and designed the experiments. DG, HL, and SM prepared experimental design of antioxidant. XY, XG, and HC prepared ALPs. YZ and JM prepared the determination of purified ALPs' structural characterization. DG and HC wrote the manuscript. All authors have read and approved the final manuscript.
